# Frequent Users of Hospital Emergency Departments in Korea Characterized by Claims Data from the National Health Insurance: A Cross Sectional Study

**DOI:** 10.1371/journal.pone.0147450

**Published:** 2016-01-25

**Authors:** Jung Hoon Woo, Zachary Grinspan, Jason Shapiro, Sang Youl Rhee

**Affiliations:** 1 Department of Biomedical Informatics, Columbia University, New York, New York, United States of America; 2 Department of Healthcare Policy and Research, Weill Cornell Medical College, New York, New York, United States of America; 3 Department of Emergency Medicine, Mount Sinai School of Medicine, New York, New York, United States of America; 4 Department of Internal Medicine, Kyung Hee University School of Medicine, Seoul, Korea; Lee Kong Chian School of Medicine, SINGAPORE

## Abstract

The Korean National Health Insurance, which provides universal coverage for the entire Korean population, is now facing financial instability. Frequent emergency department (ED) users may represent a medically vulnerable population who could benefit from interventions that both improve care and lower costs. To understand the nature of frequent ED users in Korea, we analyzed claims data from a population-based national representative sample. We performed both bivariate and multivariable analyses to investigate the association between patient characteristics and frequent ED use (4+ ED visits in a year) using claims data of a 1% random sample of the Korean population, collected in 2009. Among 156,246 total ED users, 4,835 (3.1%) were frequent ED users. These patients accounted for 14% of 209,326 total ED visits and 17.2% of $76,253,784 total medical expenses generated from all ED visits in the 1% data sample. Frequent ED users tended to be older, male, and of lower socio-economic status compared with occasional ED users (p < 0.001 for each). Moreover, frequent ED users had longer stays in the hospital when admitted, higher probability of undergoing an operative procedure, and increased mortality. Among 8,425 primary diagnoses, alcohol-related complaints and schizophrenia showed the strongest positive correlation with the number of ED visits. Among the frequent ED users, mortality and annual outpatient department visits were significantly lower in the alcohol-related patient subgroup compared with other frequent ED users; furthermore, the rate was even lower than that for non-frequent ED users. Our findings suggest that expanding mental health and alcohol treatment programs may be a reasonable strategy to decrease the dependence of these patients on the ED.

## Introduction

The National Health Insurance (NHI) has provided universal medical coverage for the entire Korean population since 1977. However, demographic and social changes in Korea have strained the finances of NHI, leading to calls for reform [1]. For example, the cost of insured medical care increased by 14%, on average, each year between 1995 and 2002 [2]. Furthermore, these financial pressures will increase with time due to the rapidly aging population [1].

Controlling healthcare spending is a complex issue not only in Korea but also in other countries, like the US [3]. Several interventions to reduce healthcare costs are under investigation; these often focus on avoidable hospital use [4]. For example, one approach to managing growing healthcare costs in those countries is to control potentially avoidable emergency department (ED) visits because, on average, an ED visit costs significantly more than an outpatient visit to a clinic or a doctor’s office [3].

Frequent ED users, operationally defined as patients with four or more ED visits per year, account for more than 20% of annual ED visits; however, these only account for 4–8% of all ED patients [5]. This subgroup of ED users is inconsistently characterized and understudied. In a previous study, frequent ED use was associated with younger age and lower-acuity visits [6]. However, a review of 25 studies conducted in the US suggested that frequent users were more likely to be older, sicker, and in need of better care [5, 7–9]. These mixed results suggest that frequent ED users are a heterogeneous population [5, 10–12]. Previous studies conducted in one or a few centers may not have adequately represented the overall frequent ED user population.

Reasons for frequent ED use are also understudied. Some work suggests that some people visit the ED frequently because they lack access to high quality primary care, whereas EDs are always open and accessible by public transportation and ambulance [13, 14]. However, a recent study of Medicaid patients in New York City showed that many individuals frequently visit the ED despite high rates of primary care use [15]. Understanding the characteristics of frequent ED users is critical to designing effective interventions to reduce their visits and the associated healthcare costs.

In this study, we examined the frequent ED user population in the Korean health system. We used representative data sampled from medical insurance claims provided by the Korean Health Insurance Review Agency. The data cover over 97% of the Korean population, allowing us to draw population-based conclusions.

We defined and characterized frequent ED users in the Korean health system by comparing patient features, including medical costs, mortality, treatment duration, leading diagnosis, and demographic information, with those of non-frequent users. We sought to understand whether the findings about frequent ED users in prior studies in the US healthcare system would be replicated in the Korean population, and whether these findings are independent of insurance status or ethnicity [16–18].

## Materials and Methods

### 1.1 Study setting and population

NHI is the only medical insurance operated by the Korean government. NHI is a compulsory social security system, and it covers over 97% of Korea’s population. The Korea Health Insurance Review Agency (HIRA) is a government organization that reviews and assesses medical claims for NHI and maintains a database of medical records for the entire Korean population.

Claims data for 1% of the population, collected from January 1, 2009, through December 31, 2009, were selected randomly by the HIRA, de-identified, and distributed for research.

### 1.2 Definition of ‘frequent users’

We defined frequent ED users as patients who visited the ED four or more times in a year. We used at least four visits as a threshold because it is the most common definition of ‘frequent’ ED use [5, 19].

### 1.3 Study protocol

In this study, we conducted a retrospective cross-sectional study using the claims data. The records included age, gender, weight, hospital arrival route, date of admission, days in hospitalization, surgical status, insurance type, major diagnosis codes (coded using the fifth edition of the Korean Standard Classification of Disease, ‘KCD5’), day of care, service category, payer’s amount, patient’s out of pocket cost, total amount, and diagnosis codes (KCD5) for comorbid conditions. The data also included a unique variable, ‘People for medical aid’ (‘Euryoboho Dasesangja’ in Korean), which is a public assistance program targeted at poor individuals who are recipients of the National Basic Livelihood Security System in Korea as a part of the social welfare programs [20]. Approximately 3% of Korean population were covered by ‘medical aid’ [21].

We compared means, proportions, and distributions of characteristics between frequent users and non-frequent users. We used χ^2^ tests to measure the significance of associations for categorical variables and Student’s *t*-tests for continuous variables. We used multiple logistic regression to assess the independent contribution of variables to frequent ED use. Age, gender, medical aid, treatment duration, death, number of outpatient department visits, and surgery for the primary diagnosis (binary variable) were included as covariates in the model. To evaluate relationships between the number of ED visits and the presence of certain diseases, we examined the frequency of ED use according to the percentage of people having each disease. Simple linear regression was used to test the hypothesis, and the Benjamini–Hochberg algorithm was used to control for errors due to the use of multiple tests. The ‘R’ software (ver. 2.14.1) was used to perform all statistical tests [22].

### 1.4 Ethics statement

This study was approved by the institutional review board of the Kyung Hee University Hospital (IRB No. KMC IRB 1411–08). Because this study analyzed publicly available, anonymized, and de-identified data, informed consents were waived.

## Results

### 2.1. Overview of the claims data regarding ED visits

**Fig 1:**
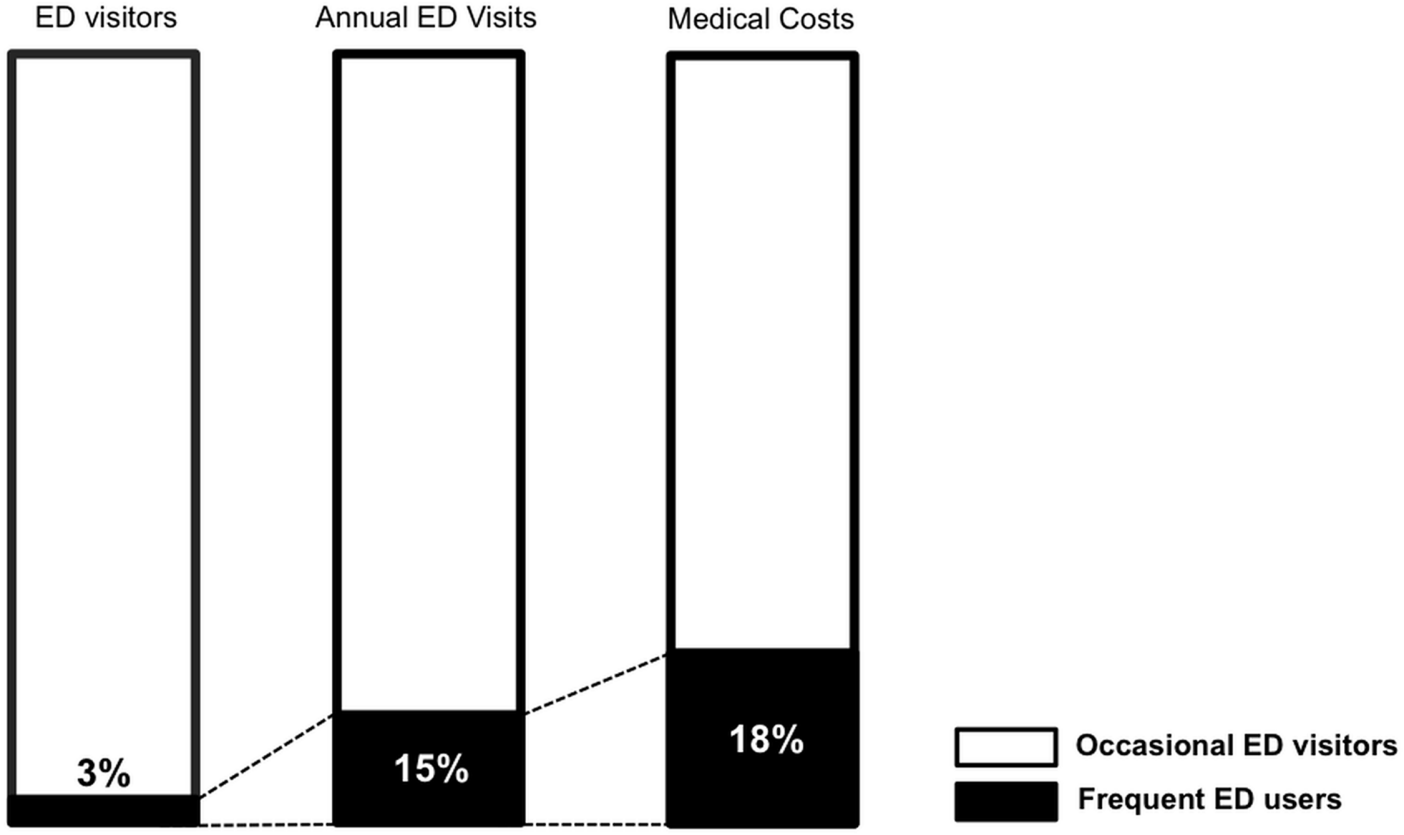
Number of visits and costs for frequent ED users. Frequent ED users accounted for about 3% of all ED users, and this 3% of users accounted for 14% of total annual ED visits and 17% of total expenses generated from the all ED visits.

We identified 209,326 claims for ED visits by 156,246 individuals in 2009. The number of ED visits per individual ranged from 1 to 26. Frequent users (4+ visits) accounted for 3.1% (4,835 patients) of all ED users. Among the 209,326 ED visits, 14.0% (29,327 visits) were by the 3.1% people classified as frequent users (Fig 1).

### 2.2. Medical costs

The total medical expense for ED visits in 2009 among this 1% sample was 480 billion Korean Won (KRW), roughly equivalent to $448 million. Frequent ED users generated expenses of 82,422,715,220 KRW ($76 million), accounting for 17% of total expenses of all ED users (Fig 1). Per visit, the medical expense associated with by patients in the frequent ED users group was 2,718,501 KRW (95% CI = 2,628,112–2,808,890) or $2,515 ($2,431–2,598), which was 30% more than the amount from patients in the occasional ED users group, 1,905,261 KRW (95% CI = 1,887,776–1,922,745) or $1,762 (1,746–1,778; p < 0.001).

### 2.3. Characteristics of frequent ED users

According to the bivariate logistic regression analysis, frequent ED use was significantly associated with older age, male gender, and lower socio-economic status (Table 1). Moreover, frequent ED users were more likely to stay longer in the hospital when admitted, to visit outpatient clinics four times more frequently in a year, to undergo a surgical procedure for the primary diagnosis, and to have increased mortality compared with occasional ED users.

**Table 1:** Characteristics of frequent and occasional ED users.

Variables	Frequent ED users (*n* = 4,835)	Occasional ED users (*n* = 151,411)	Significance of Association (*p*)
Age [95% CI]	58.8 [58.3–59.4]	46.4 [46.2–46.5]	<0.001
Gender (n,%)			
Male	2,836 (58.7)	77,980 (51.5)	<0.001
Female	1,999 (41.3)	73,431 (48.5)	
Medical Aid			
Yes	1,544 (31.9)	14,215 (9.4)	<0.001
No	3,291 (68.1)	137,196 (90.6)	
Treatment duration (days) [95% CI]	21.8 [21.5–22.0]	15.53 [15.47–15.60]	<0.001
Death			
Yes	562 (11.6)	5,328 (3.5)	<0.001
No	4,273 (88.4)	146,083 (96.5)	
Operation for Primary Diagnosis			
Yes	2,423 (50.1)	55,182 (36.4)	<0.001
No	2,412 (49.9)	96,229 (63.0)	
Outpatient Department Visits (days) / year [95% CI]	4.7 [3.9–5.5]	1.19 [1.15–1.23]	<0.001

In the multivariable logistic regression analysis, all the variables except ‘medical aid’ showed statistically significant levels of association with frequent ED use (Table 2). Increased odds of frequent ED use were associated with older age (OR = 1.008, 95% CI = 1.006–1.009, per year; p < 0.001), female gender (0.67 (0.62–0.71); p < 0.001), higher mortality (1.86 (1.65–2.08); p < 0.001), more outpatient department visits (1.056 (1.047–1.064); p < 0.001), longer treatment duration (1.036 (1.033–1.039); p < 0.001), and higher probability of undergoing an operation for the primary diagnosis (1.69 (1.57–1.80); p < 0.001).

**Table 2:** Independent risk factors of frequent ED use.

Variables	Estimated Odds Ratio	95% Confidence Interval for Odds Ratio	*p*
Age (years)	1.008	1.006–1.009	<0.001
Gender (F)	0.67	0.62–0.71	<0.001
Medical Aid (no)	5.8 e-^16^	2.7e-^121^–1.2e^-90^	0.8
Death (yes)	1.86	1.65–2.08	<0.001
Outpatient Department Visits /year	1.056	1.047–1.064	<0.001
Treatment duration	1.036	1.033–1.039	<0.001
Operation for Primary Diagnosis (yes)	1.69	1.57–1.80	<0.001

### 2.4. Acuity of patients

**Fig 2:**
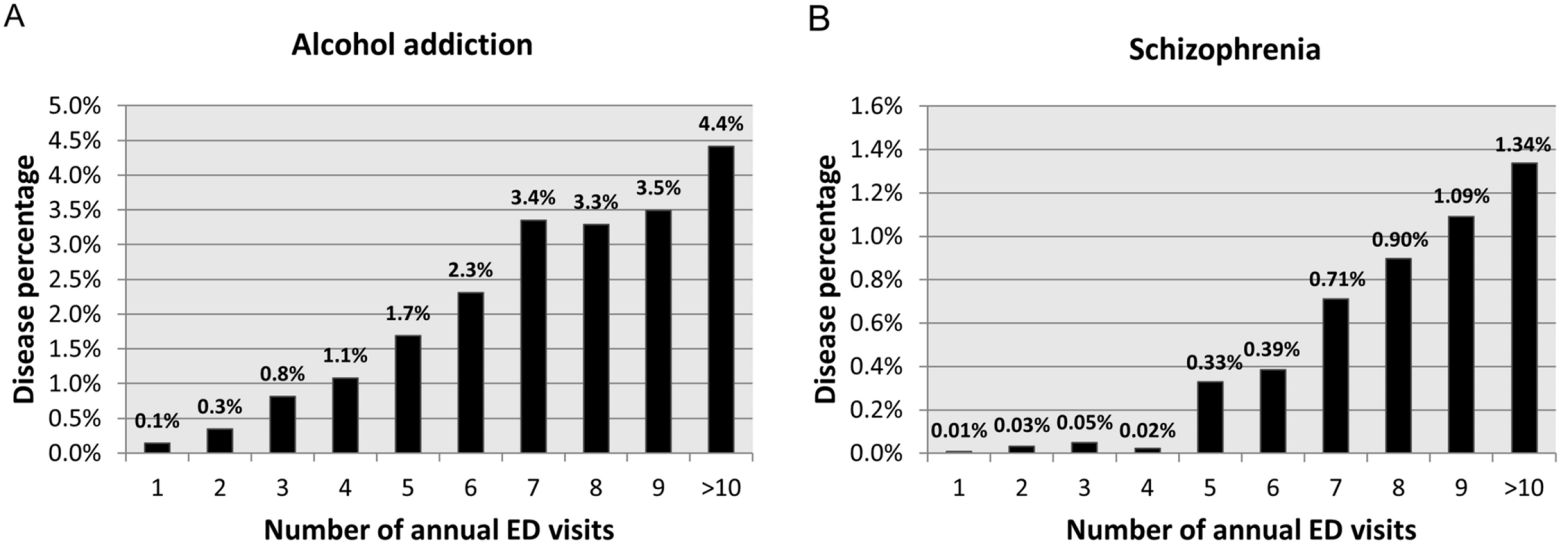
Correlations of the numbers of ED visits with (a) alcohol-related diseases and (b) Schizophrenia. Alcohol-related diseases and schizophrenia showed the strongest and second-strongest linear associations with the number of annual ED visits among 8,425 primary diagnoses.

Table 3 shows the 10 most commonly identified primary diagnoses. Alcohol addiction/chronic alcoholism and schizophrenia were among the top-five primary diagnoses in frequent ED users, but not in occasional ED users. We found a statistically significant relationship between the number of ED visits and alcohol addiction (FDR adjusted, p = 0.0008) and schizophrenia (FDR adjusted, p = 0.022; Fig 2). Among 8,425 primary diagnoses, only these two diseases passed the significance threshold (adjusted p < 0.05) after the correction for multiple testing.

**Table 3:** Top 10 primary diagnoses in frequent and occasional ED users.

Frequent ED users	%	Occasional ED users	%
*n* = 4,835 (3.1%)		*n* = 151,411 (96.9%)	
Pneumonia	6.1%	Communicable Diseases	3.7%
Alcohol addiction/Chronic alcoholism/Mental and behavioral disorders due to alcohol dependence syndrome	4.6%	Pneumonia	3.3%
Cerebral infarction	3.9%	Acute appendicitis	2.4%
Schizophrenia	3.2%	Gastroenteritis and colitis	2.0%
Hypertensive diseases	3.1%	Cerebral infarction	1.8%
Communicable Diseases	2.8%	Infectious or septic gastroenteritis hemorrhagic NOS / Acute hemorrhagic diarrhea	1.5%
Malignant neoplasm of bronchus or lung	2.8%	Acute pyelonephritis/Acute pyelitis	1.4%
Hepatocellular carcinoma	2.4%	Spontaneous vertex delivery	1.0%
Chronic kidney disease, unspecified	2.2%	Fever/ Hyperpyrexia	0.9%
Chronic kidney disease/Chronic uremia/ Diffuse sclerosing glomerulonephritis	2.2%	Asthma/ Asthmatic bronchitis	0.9%

Moreover, the subgroup of frequent ED users with alcohol addiction or schizophrenia showed unique characteristics when compared with other frequent ED users. First, this subgroup represented 3.8% of the frequent ED users in this study. They stayed longer in the hospital when compared with other frequent ED users. However, they were less likely to undergo a surgical procedure for the primary diagnosis, they had fewer annual outpatient department visits, and more importantly, there were no deaths in the subgroup, whereas we observed a 12.1% of mortality rate among the other frequent ED users (Table 4).

**Table 4:** Comparison of frequent ED users with schizophrenia or alcohol addiction with all other frequent ED users.

	Frequent ED users with Schizophrenia or Alcohol addiction	%	All other Frequent ED users	%	Significance of Association
	*n* = 186 (3.8%)		*n* = 4,649 (96.2%)		*p*
Age [95% CI]	48.3 [46.8–49.9]		59.2 [58.7–59.8]		<0.001
Gender					
Male	151	81.2%	2,685	57.8%	<0.001
Female	35	18.8%	1,964	42.2%	
Treatment duration (days) [95% CI]	27.0 [26.4–27.7]		21.6 [21.3–21.8]		<0.001
Death					
Yes	0	0%	562	12.1%	<0.001
No	186	100%	4,087	87.9%	
Operation for Primary Diagnosis					
Yes	3	1.6%	2,420	49.95%	<0.001
No	183	98.4%	2,229	50.05%	
Outpatient Department Visits (days) / year [95% CI]	2.35 [1.85–2.85]		4.82 [4.03–5.61]		<0.001

## Discussion

### Summary of findings

We found that frequent ED users accounted for 3.1% of all Korean ED patients and 14.0% of all ED visits. Regarding medical costs, 3.1% of the frequent ED users consumed more than 17% of total ED costs. The frequent ED users were more likely to be older, male in gender, and of lower socio-economic status. Frequent ED users were also “sicker” than occasional ED users. For example, the frequent users had longer treatment durations and required more surgical procedures for their primary diagnosis; importantly, their likelihood of dying during a hospital visit was much higher than that of non-frequent ED users. Furthermore, frequent ED users were also heavier users of other segments of the healthcare system (e.g., outpatient clinics) than were occasional ED users. Finally, there was a tight correlation between the frequency of ED use and the likelihood of having either schizophrenia or alcohol abuse.

### Comparison with previous studies

According to the recent systematic review, frequent ED users in the US healthcare system accounted for 4.5–8% of all ED patients, and the frequent users accounted for 21–28% of all ED visits [2]. The percentage of frequent ED users in the Korean health system was slightly lower than that in the US; however, the proportion of ED visits made by frequent ED users was similar, about four times greater than the actual proportion of ED patients. The characteristics of the frequent ED users in our study were consistent with the study recently published by Capp et al., which showed that an increased frequency of ED use was greatly correlated with older age, male gender, homelessness, and lack of a primary care provider [23]. Consistent with our findings, previous studies have found that health conditions of the frequent ED users were more severe (e.g., increased hospital admissions, higher rates of ED return visits, more comorbidities, and higher chronic illness) compared with those of occasional ED users [24, 25].

### Association between frequent ED use, alcohol addiction, and mental disorders

In our study, the frequent users were more likely to have alcohol addiction and/or schizophrenia than were occasional ED users. These two conditions appeared at the top of the list of primary diagnoses in frequent users, but not in occasional ED users. This trend has been repeatedly found in previous studies of other healthcare systems. Fuda and Immekus found higher numbers of visits related to substance abuse and mental disorders [26]. Mandelberg et al. also reported that frequent users were more likely to visit with alcohol-related illnesses [27]. More recently, Billings and Raven reported that a principal diagnosis of mental illness increased with the number of ED visits during the study year, but mental illness accounted for a relatively small share of ED visits, 3.8% in their study [15]. Missanian et al. also reported that frequent ED visitors with psychiatric complaints were more prevalent than those with other chronic conditions, such as hepatitis and HIV [28]. Curran et al. also pointed out that psychiatric and substance use problems are commonly found to be contributing factors to frequent ED use [29]. This trend and the percentage share were consistent with our results. Finally, a recent review of avoidable hospital use suggested that health services interventions should focus on individuals with mental illness and substance abuse, in agreement with our findings [30].

### Differential severity in the frequent ED users

We found that the subgroup of frequent ED users with alcohol addiction had unique characteristics. They appeared to be less ill than other frequent ED users; for example, mortality, annual outpatient department visits, and frequency of undergoing surgical procedures were much lower in this group than in other frequent ED users, and they were even lower those for than for non-frequent ED users. Thus, the patients in this subgroup visited the ED frequently even though they did not have urgent medical problems.

### Limitations

In this study, we characterized frequent ED users using a population-based nationally representative data set, which yielded results consistent with previous studies. However, some limitations should be mentioned. First, the data were from a randomly selected 1% sample of all medical records of the Korean population, so the sampling may under-represent some small subgroups that may also be part of the frequent ED population (even if the selection was designed to include representative data points). Second, this was a cross-sectional study, limiting any causal inference. Third, although our work points to one population that may benefit from improved out-of-hospital health services, we recognize that lowering health care costs in Korea will require a multi-faceted, complex, and comprehensive set of reforms to the Korean Health System. Finally, follow-up studies are required to translate and to confirm our initial findings. For example, future work using a retrospective cohort study design would be feasible to estimate causal effects of the predictors on frequent ED uses, assuming that HIRA opens the claims data for further consecutive years.

### Overall conclusion and clinical implications

According to the present study, frequent ED users in Korea were responsible for a substantial proportion of medical costs, as has been shown in other healthcare systems. However, frequent ED users’ greater use of this facility does not seem easily amenable to change because subjects in this subgroup generally had more severe health conditions compared with occasional ED users. However, we also identified a unique subgroup of the frequent ED users who may not have urgent medical problems compared with other frequent users. This may represent a population with potentially avoidable costs in the Korean healthcare system. Our findings suggest that interventions should focus on more robust outpatient management of alcohol addiction and schizophrenia before these patients visit the ED to reduce the overuse of emergency services.
